# Macrophage Ontogeny, Phenotype, and Function in Ischemia Reperfusion-Induced Injury and Repair

**DOI:** 10.34067/KID.0000000000000376

**Published:** 2024-02-01

**Authors:** Bibi Maryam, Morgan E. Smith, Sarah J. Miller, Hariharasudan Natarajan, Kurt A. Zimmerman

**Affiliations:** 1 Division of Nephrology, Department of Internal Medicine, University of Oklahoma Health Sciences Center, Oklahoma City, Oklahoma; 2 Department of Internal Medicine, University of Oklahoma Health Sciences Center, Oklahoma City, Oklahoma

**Keywords:** acute kidney failure, AKI

## Abstract

AKI is characterized by a sudden, and usually reversible, decline in kidney function. In mice, ischemia–reperfusion injury (IRI) is commonly used to model the pathophysiologic features of clinical AKI. Macrophages are a unifying feature of IRI as they regulate both the initial injury response as well as the long-term outcome following resolution of injury. Initially, macrophages in the kidney take on a proinflammatory phenotype characterized by the production of inflammatory cytokines, such as CCL2 (monocyte chemoattractant protein 1), IL-6, IL-1*β*, and TNF-*α*. Release of these proinflammatory cytokines leads to tissue damage. After resolution of the initial injury, macrophages take on a reparative role, aiding in tissue repair and restoration of kidney function. By contrast, failure to resolve the initial injury results in prolonged inflammatory macrophage accumulation and increased kidney damage, fibrosis, and the eventual development of CKD. Despite the extensive amount of literature that has ascribed these functions to M1/M2 macrophages, a recent paradigm shift in the macrophage field now defines macrophages on the basis of their ontological origin, namely monocyte-derived and tissue-resident macrophages. In this review, we focus on macrophage phenotype and function during IRI-induced injury, repair, and transition to CKD using both the classic (M1/M2) and novel (ontological origin) definition of kidney macrophages.

## Clinical Overview of AKI

AKI is characterized by the abrupt loss of kidney function resulting in decreased GFR, reduced urine output, and increased serum creatinine.^1^ The incidence of AKI is 10%–15% in all hospital admissions and >50% in intensive care unit patients, with AKI accounting for over two million deaths every year globally.^2,3^ There are multiple risk factors associated with worsened outcomes after AKI, including a history of diabetes, male sex, malnutrition, cirrhosis, and older age at presentation.^4–6^ Causes of AKI can be broadly grouped into three categories: prerenal, intrinsic renal, and postrenal.^7^ Prerenal AKI is caused by decreased renal perfusion (dehydration, heart failure, hemorrhage, *etc*.), whereas the cause of postrenal AKI is inadequate urinary drainage (*e.g*., kidney stones, abdominal or pelvic mass, prostatic disease).^8,9^ Intrinsic AKI is caused by direct kidney damage that is the result of acute tubular necrosis, acute interstitial nephritis, and vascular diseases such as hemolytic uremic syndrome, vasculitis, and hypertensive emergencies, among others.^10,11^ Clinically, different criteria are used to define AKI, including Risk, Injury, Failure, Loss of kidney function, and ESKD; AKI Network; and Kidney Disease Improving Global Outcomes (KDIGO); of the three, KDIGO is the one most commonly used in clinic. KDIGO defines AKI as the presence of any of the following.

1. Increase in serum creatinine by 26.5 *µ*mol/L (0.3 mg/dl) or more within 48 hours.
2. Decreased urine output with urine volume during the past 6 hours being <0.5 ml/kg per hour.
3. Within the seven prior days, 1.5 times or more increase in serum creatinine from baseline.

Complications that result from the abrupt loss of GFR include but are not limited to volume overload, acid–base disorders (most commonly metabolic acidosis), uremic complications, and electrolyte disturbances. Presently, there are no available therapies which can prevent the damage and loss of kidney function that occurs during AKI; as a result, patients who develop AKI show an increased risk of developing CKD later in life.^2^ Treatment options after AKI are limited to RRT in severe cases; however, mortality still approaches 50% in these patients.^12^

## Ischemia–Reperfusion Injury Model of AKI

The ischemia–reperfusion injury (IRI) model is used to mimic clinical ischemic injury to the kidney and involves a unilateral or bilateral clamping of the renal vessels for a defined period of time.^13^ IRI is characterized by a temporary loss of blood flow to the tissue (ischemia) followed by restoration of blood flow (reperfusion).^13^ After IRI, there is an increase in oxidative damage, endothelial dysfunction, cell death *via* apoptosis and necrosis, and immune cell accumulation, eventually resulting in reduced kidney function.^14,15^ Importantly, inflammation, which is largely driven by macrophages, is a key hallmark of the response after IRI.^16^

## Macrophages

The first description of phagocytic cells came from Elie Metchnikoff in 1883 when he observed that starfish larvae injected with thorns were quickly surrounded by white blood cells.^17^ These cells would later be termed macrophages, owing to their large size (macro) and ability to engulf foreign particles (phage). *In vivo*, the primary function of macrophages is the recognition, engulfment, and digestion of pathogens, cellular debris, and foreign substances, contributing significantly to both innate and adaptive immune response.^18,19^ Macrophages also play a pivotal role in tissue repair, inflammation modulation, and antigen presentation, making them essential contributors to immune surveillance and homeostasis.^18^ In the kidney, macrophages are involved in both the early and sustained responses to injury,^19,20^ as outlined below.

## Epithelial–Macrophage Crosstalk after Injury

After IRI, damaged and dying tubular epithelial cells (TECs) release damage-associated molecular patterns (DAMPs), also known as alarmins and danger signals, that bind to pattern recognition receptors (PRRs) on macrophages.^21^ PRRs can be expressed in intracellular (*e.g*., cytosolic, endosomal, and cellular membranes) or extracellular compartments (*e.g*., secreted forms present in interstitial fluid and bloodstream).^22–24^ PRRs consist of four subfamilies—the nucleotide-binding oligomerization domain, leucine-rich repeats containing receptors, the Toll-like receptors, the retinoic acid-inducible gene 1–like receptors (aka retinoic acid-inducible gene 1–like helicases), and the C-type lectin receptors.^25^ These various PRRs recognize DAMPs, initiating an inflammatory response that coordinates leukocyte recruitment, upregulates proinflammatory cytokine production, and shapes the overall immune response.^26^

In this review, we begin by summarizing previous literature implicating M1 and M2 macrophages in IRI. We then describe how the recent literature has shifted away from describing macrophages on the basis of M1/M2 polarization status and, instead, defines macrophages on the basis of ontological origin (*i.e*., where they come from). Finally, we finish by highlighting recent data that describe the involvement of macrophages of different ontologic origins in IRI and other mouse models of AKI.

## The M1/M2 Macrophage Paradigm

It has long been accepted that macrophages are highly plastic cells that can adapt their phenotype and function to meet the ongoing challenges in the tissue. For example, *in vitro* studies demonstrated that macrophages can polarize into a proinflammatory (M1) or anti-inflammatory (M2) phenotype depending on the cytokine environment.^27^ *In vitro* treatment of bone marrow–derived macrophages with lipopolysaccharide and IFN-*γ* results in a proinflammatory M1-like macrophage phenotype, whereas glucocorticoids, immune complexes, and IL-4/IL-13 induce an anti-inflammatory, M2-like phenotype.^27^ Each macrophage phenotype is associated with hallmark cytokine production; TNF*α*, IL-1*β*, inducible Nitric Oxide Synthase, and IL-6 for M1 macrophages, and TGF-*β*, IL-10, ARG1, and CD206 for M2 macrophages.^27^ Although it is important to highlight that M1/M2 polarization is a byproduct of *in vitro* cell culture conditions and macrophages *in vivo* often express markers associated with both M1 and M2 macrophages,^28,29^ the take-home message from these studies is that M1 macrophages have a proinflammatory phenotype,^30–34^ while M2 macrophages have a proreparative phenotype.^35^ Thus, it is believed that M1 macrophages play an important role in the early phase of AKI by removing dead and damaged cells, activating other immune cells *via* inflammatory cytokine production, and phagocytizing cell debris. By contrast, M2 macrophages produce anti-inflammatory and reparative cytokines, such as fibroblast growth factor, TGF-*β*, and vascular endothelial growth factor, which can drive both epithelial repair and progression to CKD, depending on the time in tissue.^36^ Below, we discuss the M1 and M2 driven proinflammatory and anti-inflammatory pathways involved in IRI *in vivo*, as well as the functional importance of each population in response to IRI.

## M1 Macrophage (Proinflammatory) Pathways

### Nuclear Factor Kappa-Light-Chain-Enhancer of Activated B Cells Pathway

**Figure 1:**
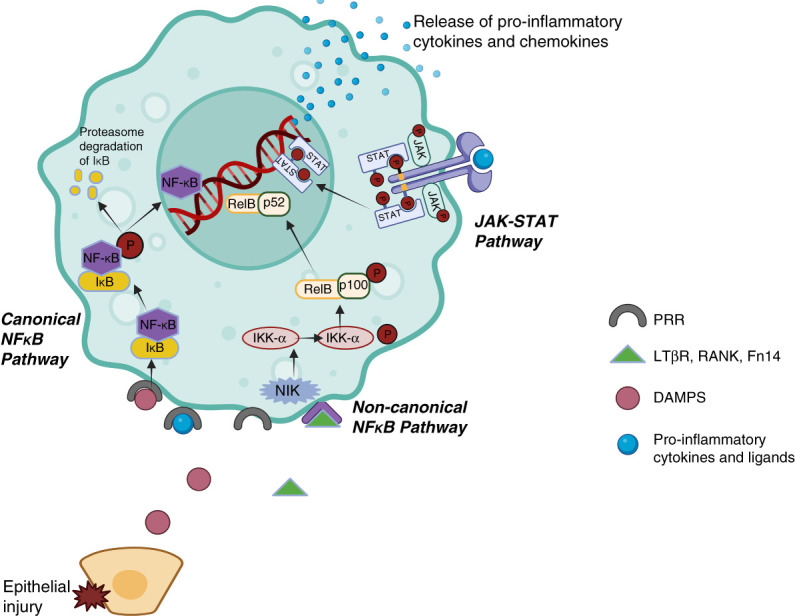
**The NF-κB and JAK-STAT pathways are increased in proinflammatory macrophages.** DAMP, damage-associated molecular pattern; JAK-STAT, Janus kinase/signal transducer and activator of transcription; LT*β*R, lymphotoxin-*β* receptor; PRR, pattern recognition receptor; RANK, receptor activator for nuclear factor κB.

After IRI, proinflammatory macrophages promote kidney damage by activating a number of transcription factors, including nuclear factor kappa-light-chain-enhancer of activated B cells (NF-κB).^37^ In its inactive state, NF-κB is sequestered in the cytoplasm by the inhibitory κB (IκB) proteins.^38^ NF-κB can be activated by two main pathways: the canonical (classic) and noncanonical (alternative) pathways.^39^ Pathogen associated molecular patterns (such as lipopolysaccharide) and proinflammatory cytokines (TNF*α*, IL-1*β*) can initiate the canonical pathway *via* binding to cell surface receptors, including PRRs, resulting in phosphorylation of the IκB kinase (IKK) (Figure 1). IKK activation leads to phosphorylation and subsequent ubiquitin-mediated degradation of IκB proteins, resulting in nuclear translocation of NF-κB and associated family members. P50/NF-κB1-c-Rel, p50/NF-κB1-p65/RelA, and less commonly P65/RelA, p50/NF-κB1, and c-Rel are canonical NF-κB signaling members.^40^

By contrast, B-cell activating factor receptor, lymphotoxin-*β* receptor, fibroblast growth factor-inducible factor-14 (Fn14), and receptor activator for nuclear factor κB are the main activators of the noncanonical pathway (Figure 1).^41^ After receptor stimulation, NF-κB inducing kinase is activated, which phosphorylates IKK*α*, thus leading to phosphorylation of p100 and degradation of its IκB-like C terminal.^42^ This results in translocation of the noncanonical dimer complex p52/NF-κB2-RelB to the nucleus.^43^

After nuclear translocation, NF-κB induces transcription of a wide variety of cytokines (TNF-*α*, IL-1, IL-2, IL-6), chemokines (CCL2, CCL3, CXCL8, CCL5), acute phase reactants (serum amyloid A), inducible effector enzymes (cyclooxygenase-2 and inducible nitric oxide synthase), and adhesion molecules (vascular cell adhesion molecule 1, intercellular adhesion molecule 1, and E-selectin) that drive the inflammatory process.^38,44,45^ It is likely that this is a critical pathway during the acute phase of injury^46^ as the inhibition of NF-κB signaling decreases the inflammatory response and fibrosis in the kidney after IRI.^47,48^

### Janus Kinase/Signal Transducer and Activator of Transcription Pathway

The Janus kinase/signal transducer and activator of transcription (JAK-STAT) signaling pathway is an intracellular signal transduction pathway that consists of three main components: signal transducer and activator of transcription 1–6, tyrosine kinase–associated receptors, and Janus kinases (JAK1, JAK2, TYK2, and JAK3).^49^ The JAK-STAT pathway can be induced by cytokines, including IL-3, IL-5, granulocyte-macrophage colony stimulating factor and reactive oxygen species.^50^ Upon a ligand binding to its cognate receptor, the phosphorylation of a tyrosine residue on bound transcription factors (STATs) by the receptor-associated cytosolic tyrosine kinases (JAKs) results in nuclear translocation and gene transcription augmentation (Figure 1).^51,52^ In IRI, the activation of the JAK-STAT signaling pathway induces transcription of multiple proinflammatory cytokines.^53^ The JAK/STAT signaling pathway is also responsible for the recruitment of leukocytes^54^ and exacerbates renal injury by stimulating macrophages to secrete cytokines, such as TNF-*α*, IL-6, and IL-1*β*.^54^ It may be possible to target the JAK-STAT pathway in macrophages to reduce IRI-associated kidney damage. For example, inhibition of the JAK/STAT signaling cascade has been shown by several groups to reduce kidney injury and improve phenotypic outcome after IRI.^54–56^

### Effect of Macrophage Depletion during the Acute Phase of Injury

On the basis of their production of proinflammatory cytokines, it was predicted that genetic or pharmacologic blockade or depletion of proinflammatory M1-like macrophages would reduce IRI-induced kidney damage. In agreement with this hypothesis, depletion of macrophages before IRI with liposomal clodronate (LC), an encapsulated bisphosphonate that induces apoptosis upon phagocytic uptake,^57–60^ significantly reduced kidney damage.^61–66^ The improved phenotypic outcome after IRI was associated with reduced proinflammatory cytokine production. Importantly, reintroduction of proinflammatory macrophages to macrophage-depleted mice resulted in worsened kidney phenotype,^63^ providing conclusive evidence that proinflammatory macrophages, which accumulate during the acute phase of injury, promote disease progression. One interesting observation from the study of proinflammatory macrophages in IRI is data showing that the depletion of macrophages with LC, but not using CD11b-DTR or CD11c-DTR mice, significantly improved kidney damage.^65,66^ This occurred despite the fact that all models had a similar reduction in kidney macrophage number. Instead, the authors hypothesized that the failure to rescue the kidney phenotype in CD11b-DTR and CD11c-DTR mice compared with LC-treated mice was mainly driven by differential effects on proinflammatory cytokines, such as IL-6 and monocyte chemoattractant protein 1.^66^ These data suggest that additional macrophage heterogeneity is present in the kidney and that each depletion method might differentially affect macrophage subsets.

## M2 Macrophage (Proreparative) Pathways

### ARG1

After injury, exposure to dead cell debris leads to increased granulocyte-macrophage colony stimulating factor expression by outer medullary epithelial cells, prompting increased expression of Arginase-1 (*Arg1*) by adjacent macrophages.^67,68^ *Arg1*, which is responsible for conversion of arginine into ornithine and eventually polyamines, is involved in the repair process through secretion of a yet unidentified factor or factors that promote tubular cell proliferation in the kidney after injury.^69,70^ In mice, loss of *Arg1* expression in macrophages was associated with decreased tubular cell proliferation, slowed repair, worsened BUN, increased creatinine, and significantly reduced survival after AKI.^67^ They also found that creatinine and BUN values were not different between groups 1 day after injury, suggesting that arginase-1 is specifically required for macrophage-dependent repair after IRI.

### Wnt-*β* Catenin Pathway

**Figure 2:**
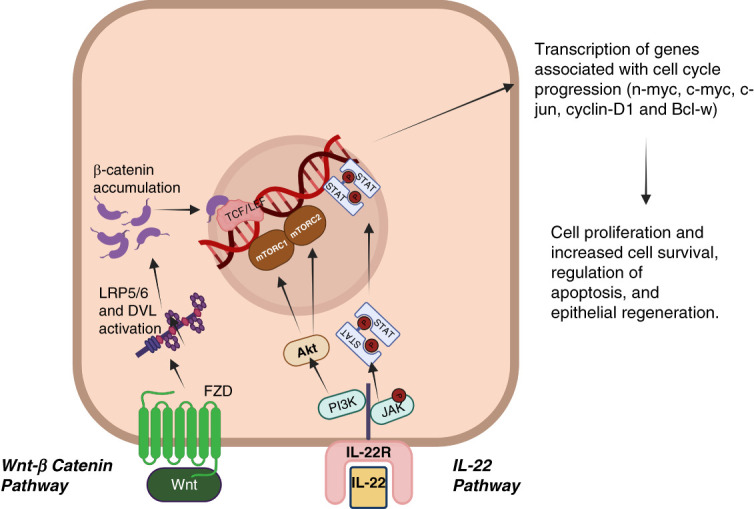
**Macrophage-derived IL-22 and Wnt drive epithelial repair after injury.** DVL, disheveled protein.

Macrophages promote tubular repair *via* the production of Wnt ligands.^71^ In healthy kidneys, canonical Wnt-*β*-catenin pathway activity is restricted to the renal papilla and the transcription of downstream *β*-catenin genes is repressed by a destruction complex. By contrast, after injury, the Wnt-*β*-catenin pathway is activated in both the cortical and medullary region of the kidney.^71^ Extracellular Wnt ligands bind to Frizzled protein (FZD), activating low-density lipoprotein receptor-related proteins (LRP5/6), and disheveled protein.^72^ Disheveled protein then disrupts the destruction complex, causing *β*-catenin accumulation in the cytoplasm and, ultimately, nuclear translocation (Figure 2). On entering the nucleus, *β*-catenin activates the t-cell factor/lymphoid enhancer factor transcription complex, mediating the transcription of genes associated with cell–cycle progression (n-myc, c-jun, c-myc, cyclin D1, and Bcl-w) and epithelial regeneration.^72–75^ In macrophages, deletion of *Wnt7b* in colony-stimulating factor-1 receptor expressing macrophages resulted in an impaired reparatory response after IRI,^71^ suggesting this pathway plays an important role in resolving damage in the kidney after injury. In agreement with this hypothesis, data from our laboratory showed that kidney resident macrophages (KRM) isolated from mice undergoing IRI had increased the expression of Wnt-associated genes,^76^ once again suggesting a functional role for Wnt signaling after injury.

### IL-22

IL-22 plays an important role in promoting recovery after IRI by promoting TEC proliferation.^77,78^ In IRI, IL-22 is predominantly expressed by mononuclear cells, including dendritic cells and macrophages, whereas the IL-22R is expressed by multiple cell types, including tubular epithelium.^79,80^ Upon ligand/receptor interaction, downstream signaling is initiated through the Jak1/Stat1/3/5 signaling cascade. IL-22R engagement also induces the activation of PI3K/Akt and mitogen-activated protein kinase pathways.^79^ Using an *in vivo* system of TEC regeneration, Xu *et al.* injected mice with adenovirus expressing IL-22 intravenously 5 days before renal IRI. They showed that increased IL-22 resulted in accelerated epithelial cell growth and improved kidney function after IRI, as measured by higher levels of serum creatinine and BUN in the control group as compared with IL-22 administered mice.^78^ The authors proposed that protective effects of IL-22 were dependent on the activation of AKT and STAT3 in the proximal TECs (Figure 2).^78^ They also observed increased Bcl-2, a key regulator of apoptosis, after treatment with IL-22, suggesting an additional mechanism through which this cytokine could exert a reparative effect. Similarly, IL-22 depletion studies showed that reduced levels of IL-22 leads to impaired epithelial repair and functional recovery after AKI.^80^

### Effect of Macrophage Depletion on the Reparative Phase of Injury

Macrophages play an important role in the reparative phase after IRI.^81,82^ To test the functional importance of macrophages during the recovery phase after IRI, Lee and colleagues subjected mice to unilateral IRI and contralateral nephrectomy followed by LC-mediated macrophage depletion 48 and 72 hours after injury.^63^ Importantly, this approach did not affect the initial recruitment of inflammatory monocytes to the kidney during the acute phase of injury. LC-treated mice exhibited substantial diminution of tubule regeneration with fewer epithelial cells compared with the controls.^63^ BUN values were indistinguishable in both groups during the initial phase; however, macrophage-depleted mice exhibited less improvement in GFR at days 5 and 7 after injury.^63^ In agreement with these data, data collected from several other groups showed that depletion of M2-like macrophages, which accumulate during the reparative phase, resulted in failed tubular repair, worsened kidney function, and increased interstitial fibrosis.^63,83–85^ While most data suggest that M2-like macrophages promote repair after injury, data from other groups showed that depletion of M2-like macrophages improved histology, reduced kidney injury, and improved fibrosis outcomes.^86–88^ Thus, the exact role of M2-like macrophages in IRI-induced kidney damage warrants further investigation.

### Kidney Fibrosis and AKI to CKD Transition

Fibrosis is the process whereby normal renal tissue is replaced by extracellular matrix (ECM). On the basis of the published data, it is hypothesized that macrophages play a profibrotic role in the kidney as depletion of macrophages resulted in reduced renal fibrosis.^89^ However, as mentioned above, the ability of macrophages to promote fibrosis after IRI is still controversial. There are two proposed mechanisms through which macrophages may promote fibrosis: the persistent accumulation of proreparative macrophages that produce profibrotic cytokines, such as TGF-*β*1,^90^ and the inability of macrophages to switch from the proinflammatory to the proreparative phenotype.^91^ Both of these processes lead to progressive renal inflammation and excessive deposition of ECM.

### TGF-*β*1

**Figure 3:**
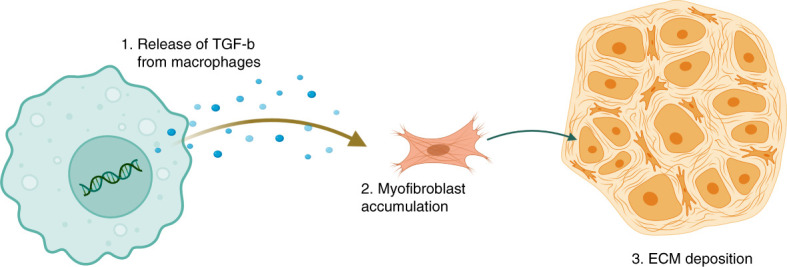
**TGF-*β* released from macrophages drives myofibroblast accumulation and ECM production after IRI.** ECM, extracellular matrix; IRI, ischemia reperfusion injury.

The TGF-*β* pathway is the main signaling pathway that is involved in fibrosis; TGF-*β* is thought to be mainly produced by macrophages^92,93^ and signals through two major receptors, TGF-*β* RII (T*β*RII) and TGF-*β*RI (T*β*RI).^94^ On binding to its receptors, TGF-*β* induces the downstream phosphorylation of Smad2/3, which forms a complex with Smad4, to induce transcription of TGF-*β* target genes, including collagen and fibronectin.^95^ Macrophages are the major producers of TGF-*β* after IRI.^96^ Excessive production of this cytokine over time drives the accumulation of myofibroblasts, cells that deposit large amounts of ECM proteins, including collagens (Figure 3).^97,98^ The exact precursor cell that gives rise to myofibroblasts is not well understood, although it has been proposed that they may arise from epithelial to mesenchymal transition, endothelial to mesenchymal transition, fibrocyte recruitment, pericyte recruitment, and resident fibroblast recruitment.^99^ TGF-*β* pathway activation also results in activation of the NLRP3, Smad3, and Caspase-1 stimulators, which further exacerbate renal fibrosis.^100^ As a result, an imbalance develops between ECM synthesis and degradation, promoting deposition of ECM components in the kidney, glomerular sclerosis, and reduced renal function.^101^ Despite a significant amount of literature pointing toward macrophage-derived TGF-*β* being a major driver of fibrosis, data indicate that genetic deletion of *Tgfb1* in myeloid lineage cells had minimal impact on fibrosis after severe IRI.^96^ Thus, there are likely multiple profibrotic cytokines produced by macrophages that drive fibrosis and CKD transition after IRI.

## Kidney Macrophage Classification On the Basis of Recent Literature

Recent literature has highlighted the fact that macrophages isolated *in vivo* express genes associated with both M1 and M2 macrophages.^76^ Thus, it has become evident that the original description of macrophages as M1 or M2, which was largely driven by *in vitro* data, does not fully recapitulate the heterogeneity of these cells *in vivo*. As such, recent literature favors the identification of macrophages on the basis of their ontological origin. For the purpose of this review, we will begin by defining kidney macrophage subsets in the mouse on the basis of ontologic origin. We will then briefly comment on how data from the mouse may correlate to humans.

**Figure 4:**
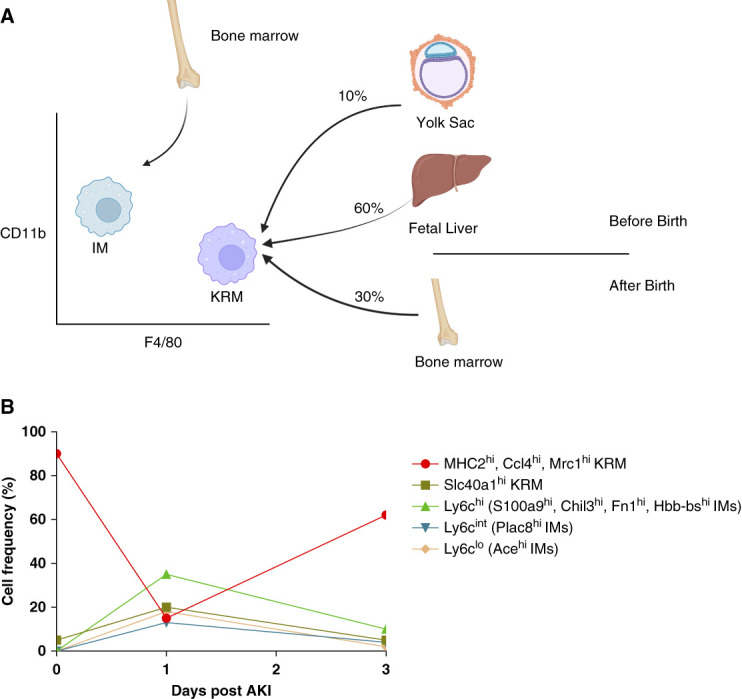
**Kidney macrophage ontogeny during development and after ischemia reperfusion injury.** (A) Macrophages originate from the yolk sac, fetal liver, and bone marrow. (B) Summary of macrophage number, grouped by ontological origin, at different time points after AKI. KRM, kidney-resident macrophages.

In the mouse, it was initially believed that all macrophages originated from bone morrow monocytes.^102,103^ However, several paradigm shifting studies have shown that macrophages in all tissues are derived both from bone marrow monocytes and embryonic precursors.^104,105^ In these studies, the authors identified two distinct macrophage populations that were present in almost all tissues: monocyte-derived macrophages and tissue-resident macrophages.^104–106^ Monocyte-derived infiltrating macrophages (IM) originate from adult bone marrow monocytes, express high levels of CD11b and intermediate levels of F4/80 (CD11b^hi^, F4/80^lo^), and are rapidly turned over throughout the animal's lifespan (Figure 4A).^107^ By contrast, tissue-resident macrophages are mainly derived from embryonic precursors, express intermediate levels of CD11b and high levels of F4/80 (CD11b^lo^, F4/80^hi^), and largely maintain their population through self-proliferation (Figure 4A).^108–110^

From the start of kidney development and throughout life, KRM derive from progenitors in waves. For example, the first wave of KRM precursors entering the kidney come from yolk sac–derived erythromyeloid (CD45^+^CD11b^lo^F4/80^hi^Ly6C^−^ cells) progenitors (EMPs) at approximately embryonic day (E) 12.5.^111^ Between days E13.5 and E16.5, fetal liver monocyte-derived KRM precursors begin entering the developing kidney and increase progressively, with their numbers rapidly exceeding those of yolk sac–derived macrophages by E16.5.^111,112^ It is still not clear whether these fetal liver monocytes come from aorta–gonad–mesonephros hematopoietic stem cells (HSCs), EMPs, or a combination of both.^107,111,112^ Finally, after birth, a third wave of KRM precursors derived from bone marrow HSCs begin to enter the kidney and engraft into the KRM niche; this process continues until the mice reach adulthood (approximately 12 weeks).^113^ Thus, KRM found in adult mice are derived from a combination of EMP (approximately 10%), fetal liver monocytes (approximately 60%), and adult, bone marrow–derived HSCs (approximately 30%).^104,113,114^

Despite extensive research in mice, there have been significant limitations preventing the identification of similar macrophage subsets in humans. For example, the main marker used to identify mouse-resident macrophages, F4/80, is also expressed by human eosinophils,^115^ although these findings have recently been called into question.^116^ Thus, until recently, it has not been possible to identify evolutionarily conserved macrophage populations across species. This gap in knowledge was recently addressed in a publication by our laboratory.^117^ In this study, we performed single-cell RNA sequencing (scRNAseq) on macrophages isolated from the kidney of adult mice, rat, pig, and humans. Using this approach, we identified a novel list of mouse KRM specific genes, including *C1qa*, *C1qb*, *C1qc*, *Cd74*, and *Cd81*, and showed that several of these genes were expressed in a single cluster of immune cells isolated from rat, pig, and human kidney tissue. We went on to validate that a single cluster of CD45^+^ immune cells from mouse, rat, and human kidney tissue expressed several of these proteins *via* flow cytometry. Furthermore, we showed that we could use our new KRM markers to identify a population of cells in the rat that received minimal input from peripheral monocytes, a hallmark of resident macrophages in the mouse.^118^ Thus, our studies identified an evolutionarily conserved KRM signature (*C1qa*, *C1qb*, *C1qc*, *Cd81*, *Cd74*) that was present in KRM across multiple species, including humans. The markers identified in our studies were independently confirmed in a separate study.^119^ In summary, there are two major populations of macrophages present in the kidney (IM, KRM) with distinct ontologic origins. Furthermore, our scRNAseq data indicate that both populations are evolutionarily conserved across multiple mammalian species.

## How Macrophages of Different Origins Respond after IRI

Recently, new studies have shed light on the behavior of KRM and IM subsets during AKI.^76,120–122^ To understand how macrophages of each origin respond to AKI, Yao and colleagues performed scRNAseq on CD11b and F4/80 positive cells isolated from the kidneys of mice 1 or 3 days post 45 minutes of unilateral IRI.^121^ Analysis of combined scRNAseq at all time points revealed four clusters of KRM, including MHC2^hi^, Ccl4^hi^, Mrc1^hi^, and Slc40a1^hi^ subsets. Analysis of cluster composition revealed a significant reduction in the number of MHC2^hi^, Ccl4^hi^, Mrc1^hi^ KRM subsets 1 day postinjury; the number of all three subsets partially rebounded toward control levels on day 3 (Figure 4B).^121^ By contrast, the number of Slc40a1^hi^ KRM increased day 1 postinjury followed by their subsequent reduction 3 days postinjury (Figure 4B). In homeostasis, KRM were involved in antigen processing and presentation (MHC2^hi^, Ccl4^hi^ subsets), myeloid cell migration and differentiation (Ccl4^hi^, Mrc1^hi^ subsets) and response to metal ions (Slc40a1^hi^). One day after injury, phagocytosis (MHC2^hi^ subset), inflammatory (Ccl4^hi^ subset), anti-inflammatory (Mrc1^hi^ subset), and wound repair (Mrc1^hi^ subset) functions were enhanced in KRM. Slc40a1^hi^ KRM mainly expressed angiogenesis and wound repair genes, including *Vcam1*, *Igf1*, and *Tnfaip2* on day 3.

The authors also identified six clusters of monocyte‐derived IMs, including four clusters of Ly6c^hi^ IMs (S100a9^hi^, Chil3^hi^, Fn1^hi^, and Hbb-bs^hi^), one Ly6c^int^ IM cluster (Plac8^hi^), and one cluster of Ly6c^lo^ IMs (Ace ^hi^).^117,121^ Analysis of cluster abundance indicates that all IM subsets increased in frequency on day 1, followed by a reduction in number on day 3 after IRI (Figure 4B). Ly6c^hi^ IMs functions were associated with clearance of apoptotic cells and maintenance of the acute inflammatory response. Ly6c^lo^ IM functions were associated with maintaining endothelial stability and regulating vasculogenesis. KRMs and IMs exhibited a similar ability to activate leukocytes and phagocytose cells after injury. Collectively, these data suggest that the ontologic origin of macrophages influences their function with KRM being involved in antigen processing and presentation, phagocytosis, inflammation, and would repair. By contrast, IMs were involved in driving the acute inflammatory response, maintaining endothelial stability, and regulating vasculogenesis.

## Function of KRM and IM during IRI

Despite data indicating the IM and KRM number and subsets are altered after IRI, there are limited data on the specific role of these subsets in disease progression, mainly owing to the inability to specifically target these cells. To tease out the function of IM and KRM in IRI, Park and colleagues took advantage of IM and KRM niche filling kinetics after LC injection.^123^ In these studies, the authors showed that KRM numbers were unchanged 72 hours post-IRI, whereas IM numbers were substantially increased,^123^ similar to the scRNAseq data described above.^121^ The authors then performed IRI in LC treated mice 2 weeks postdepletion, a time point at which KRM numbers were reduced while IM numbers were unaffected. Using this approach, the authors found that depletion of KRM resulted in worsened tubular injury and increased terminal deoxynucleotidyl transferase–mediated digoxigenin–deoxyuridine nick-end labeling positive cells suggesting that KRM promote tubular repair.^123^ In agreement with these data, depletion of KRM increased fibrosis in a renal artery stenosis model of ischemic kidney injury.^124^ The protective effect of KRM in this model was due to the production of proangiogenic and reparative factors by KRM, including *Il10*, *Arg2*, *Tgfb2*, and *Tgfbr3*.^124^ Additional data using a CD169-DTR mouse to deplete KRM support the idea that KRM play a protective role in IRI.^125^ Loss of CD169^+^ KRM was associated with increased inflammation, increased expression of *Icam1*, and worsened kidney function.^125^ Data from our laboratory further support the idea that KRM protect from IRI-induced kidney damage as KRM that accumulated after IRI injury had increased expression of genes associated with repair after injury, including Wnt family genes.^76^ Of interest, the transcriptional signature of KRM after injury mirrored that of KRM isolated from postnatal day 7 (P7) mice suggesting that dedifferentiation of KRM may promote epithelial repair and functional recovery after IRI.^76^ How the dedifferentiated KRM identified by our group corresponds to the KRM subsets identified *via* scRNAseq is unknown. In addition, data from these studies suggest that KRM have additional functions after IRI beyond production of reparative factors; these include phagocytic capacity to remove dead and dying epithelial cells, activation of the adaptive immune system, recruitment of additional immune cells, and production of proangiogenic factors that promote vessel repair after injury. Of interest, a recent study by Cheung *et al.* showed that injury changes the spatial distribution of KRMs. In this study, the authors identified seven distinct KRM subpopulations using scRNAseq, flow cytometry, and spatial transcriptomics and observed that after injury, the original localization of each subpopulation was disrupted and was not restored until at least 28 days after IRI.^120^ Once again, how these subsets correspond to previous data remain uncertain.

Additional data supporting a protective role for KRM after IRI come from studies focused on the CSF1/CSF1R signaling axis. Importantly, CSF1 signals through the CSF1R receptor expressed on KRM to drive cell survival and proliferation^126,127^; thus, it may be extrapolated that studies targeting the CSF1/CSF1R signaling axis were mainly focused on KRM. Treatment of mice with CSF1 beginning 3 days postinjury reduced tubular damage and improved kidney function when analyzed day 7.^128^ Moreover, the improvement in kidney function after CSF1 treatment was associated with increased expression of reparative cytokines, including *Wnt7b* and *Igf1*.^128^ Similarly, Zhang and colleagues showed that genetic or pharmacologic inhibition of CSF1 signaling reduced macrophage proliferation, increased inflammatory gene expression, and worsened kidney function after IRI.^129^ While the protective effect of CSF1/CSF1R signaling after IRI likely involves macrophages, one study proposed that CSF1 could directly signal to the injury tubular epithelium to drive tissue repair.^84^ However, selective deletion of epithelial cell–derived CSF1 prevented accumulation of reparative macrophages after IRI, delayed functional and structural recovery, and increased tubulointerstitial fibrosis.^130^ Thus, it is likely that the protective effects of the CSF1/CSF1R signaling axis after IRI are dependent on KRM.

The involvement of IM in IRI is clearer due to studies using *Ccr2* deficient mice, which are unable to mobilize Ly6c^hi^ monocytes out of the bone marrow and to the site of injury.^131^ In particular, *Ccr2* knockout mice were protected from IRI-induced kidney damage.^132,133^ The protective effect of IM deficiency after IRI was mainly attributable to reduced production of proinflammatory cytokines in the kidney, including IL-1*α*, IL-6, IL-12p40, and TNF-*α*.^133^

## The Role of Macrophages in Other Models of AKI

This review focuses on the role of macrophages in the renal ischemia–reperfusion model of AKI; however, macrophages are also implicated in other models of AKI. While the role of macrophages in each of these models can differ, hallmarks of their function after AKI remain the similar.^16^ For example, after the initial injury, damaged TECs release DAMPs that bind to PRR on innate immune cells to induce phagocytosis, maturation of phagolysosomes, antigen presentation, and production of proinflammatory cytokines. In addition, IM are quickly recruited to the kidney where they produce proinflammatory cytokines, such as IL-1*β*, TNF*α*, IL-12, IL-18, and IL-23, to drive tissue damage. After resolution of the initial injury, M2-like macrophages accumulate in the kidney and promote functional recovery. Once again, prolonged accumulation of M2-like macrophages in other models of kidney injury results in fibrosis.^134–136^ Despite similarities in the pathophysiological process after injury, subtle differences in macrophage involvement are appreciated. For example, loss of IM (*via Ccr2* knockout) did not affect cisplatin-induced AKI, whereas LC treatment to deplete KRM improved phenotypic outcome.^137^ However, it should be noted that these studies used a repeated low-dose cisplatin injection to induce AKI, which may alter macrophage response and involvement when compared with mice receiving a single injury event to induce AKI. For a more comprehensive view of macrophage behavior in other models of AKI, we refer readers to several other excellent studies.^138–140^

Macrophages play a multifunctional role in AKI, affecting both the injury and repair process. The versatile role of macrophages is heavily influenced by the kidney microenvironment, and the growth factors, cytokines, and chemokines present therein. In this review, we highlighted how the understanding of macrophages, and their phenotypes and function in the kidney, has changed over time. In the past, there was a strong focus on the M1 and M2 phenotypes, whereas recent studies focus more on ontological origin and how macrophage origin relates to function. We also observed that macrophages play an important role in the repair process, and by further exploring this in future, it may be possible to harness their reparative powers to promote tubular repair and recovery of kidney function.
